# Comparative evaluation of open tray impression technique: investigating the precision of four splinting materials in multiple implants

**DOI:** 10.1186/s12903-023-03583-x

**Published:** 2023-11-08

**Authors:** Priyanka Patil, V. N.V Madhav, Abdulkhaliq Ali F. Alshadidi, Ravinder S. Saini, Lujain Ibrahim N. Aldosari, Artak Heboyan, Seyed Ali Mosaddad, Saeed Awod Bin Hassan, Saurabh Chaturvedi

**Affiliations:** 1 Department of Prosthodontics, YCMM & RDS’s Dental College and Hospital, Nanded, Maharashtra India; 2 Dental Technology Department, College of Medical Applied Sciences, King Khalid University, Abha, Saudi Arabia; 3 Department of Dental Technology, COAMS, King Khalid University, Abha, Saudi Arabia; 4 Prosthodontics Department, College of Dentistry, King Khalid University, Abha, Saudi Arabia; 5 Department of Prosthodontics, Faculty of Stomatology, Yerevan State Medical University after Mkhitar Heratsi, Str. Koryun 2, Yerevan, Armenia 0025; 6 Student Research Committee, School of Dentistry, Shiraz University of Medical Sciences, Shiraz, Iran; 7 Department of Restorative Dental Sciences “RDS“, College of Dentistry, King Khalid University, Abha, Saudi Arabia

**Keywords:** Multiple implants, Splinting materials, Acrylic resin

## Abstract

**Background:**

This study aimed to determine the relative positioning accuracy of multiple implants utilizing four distinct types of splinting materials.

**Methods:**

The purpose of this in-vitro study was to compare the precision of four splinting materials in an open tray impression technique in multiple implant situations. Based on the material used for splinting, four groups were made (*n* = 40)- Group A: Conventional Method, Group B: Prefabricated Pattern Resin Framework, Group C: Prefabricated Metal Framework, Group D: Light Cured Pattern Resin, these groups were compared with the master model. A heat-cured clear acrylic resin and a master model were constructed. A pilot milling machine drill was used to drill four parallel holes in the anterior and premolar regions, which were later labeled as A, B, C, and D positions from right to left. Then, sequential drilling was carried out, and four 3.75‑mm diameter and 13-mm long ADIN implant analogs with internal hex were placed in the acrylic model using a surveyor for proper orientation. The impression posts were then manually screwed to the implant analogs using an open tray, and they were secured to the implants using 10 mm flat head guide pins with a 15 N.cm torque. 10 Open tray polyether impressions were made, and casts were poured. Each splinting method’s distortion values were measured using a coordinate measuring machine capable of recordings in the X-, Y-, and Z-axes. Comparison of mean distances for X1, X2, and X3 was made using the Kruskal-Wallis test, and Pairwise comparison was done using Post Hoc Tukey’s Test.

**Results:**

The differences between the groups were significant when assessing the distances X1, X2, and X3 (*p* < 0.05). The comparison of deviations between the groups revealed a statistically significant difference (*p* < 0.05) for the deviation distance X3 but not for the deviation distances X1 and X2. For distance Y1, the difference between the groups was statistically significant (p0.05), but it was not significant for distances Y2 and Y3. A statistically significant difference was seen in the comparison between the groups (*p* < 0.05) for the deviation distances Y1, Y2, and Y3. The results were statistically significant for the distance Z1 comparisons, namely, control vs. Group A (*p* = 0.012), control vs. Group B (*p* = 0.049), control vs. Group C (*p* = 0.048), and control vs. Group D (*p* = 0.021), and for distance Z3 comparison for control vs. Group A (*p* = 0.033). The results were statistically insignificant for the distance Z2 comparisons (*p* > 0.05).

**Conclusions:**

All splinting materials produced master casts with measurements in close proximity to the reference model. However, prefabricated pattern resin bars splinting showed the highest accuracy among the studied techniques. The most recent splinting techniques using prefabricated metal framework and light-cure pattern resin showed similar accuracy.

## Background

A viable solution for effective prosthodontic rehabilitation is the use of dental implants. These are replacements for the roots of the extracted teeth [1]. Implants have been extensively utilized to reconstruct partially and totally edentulous arches [2]. Implant dentistry necessitates a multidisciplinary team approach that produces aesthetically appealing and biologically acceptable prostheses [3]. The Glossary of Prosthodontic Terms (GPT-9) defines osseointegration as ‘the apparent direct attachment or connection of osseous tissue to an inert, alloplastic material without intervening fibrous connective tissue [4]. Osseointegration is multifactorial, depending on the precision of surgical and restorative techniques, soft tissue management, and the patient’s general and oral health [5]. It has been shown that surface morphology, topography, roughness, chemical composition, surface energy, chemical potential, strain hardening, the presence of impurities, thickness of titanium oxide layer, and the presence of metal and non-metal composites have a significant influence on bone tissue reactions [6].

Implant prostheses require a passive fit to the underlying structures [7]. Any clinically visible mismatch of the framework to osseointegrated implants can potentially create internal strains in the prosthesis framework, implants, and bone around the implant [8]. Recording the implant in a three-dimensional orientation is very necessary and crucial. A good-fitting prosthesis requires a precise impression that impacts the definitive cast and the laboratory procedure. An inaccurate impression might cause a misfit of prosthesis and mechanical or biological issues [9].

A precision fit is required because of the implant-bone connection. A natural tooth can move up to 100 μm in its periodontal ligament, allowing for some misfit to a fixed partial denture; however, an osseointegrated implant can only move ten µm [10]. Dental implant impression procedures typically fall into one of two categories: direct (open trays) or indirect (closed trays). In the open-tray method, the impression post is attached to the fixture after being placed in the mouth, and the tray containing the set impression is removed [9]. The impression coping is retained in the mouth in the closed tray technique when the set impression is removed. Splinting materials comprise acrylic resin, dental plaster, bite registration silicone, and polyether (PE). Shankar et al. [9] underlined the significance of intraoral splinting impression copings before making an impression to ensure maximum accuracy. The precision of a splinted impression method also depends on the splint’s ability to maintain its shape when subjected to the pressure of the impression [11].

The accurate replication of implant positions and the precision of impression techniques are crucial in implant dentistry. Achieving optimal fit and accuracy in implant-supported restorations depends on various factors, including the choice of impression technique and splinting materials [11].

The open tray impression technique involves capturing implant positions using impression copings. It offers advantages such as easier access, better control over impression material flow, and reduced risk of distortion during impression procedures. However, the choice of splinting materials can potentially impact the accuracy and fit of implant restorations [10]. Splinting several implants may not always provide predictable effects. Several studies have reported impression-transfer techniques, especially with regard to the benefits of either splinting transfer [12]. The rigid union of the transfers will theoretically improve the cast’s accuracy. Still, it can be observed in the literature that this step is generally insufficient to ensure that the accuracy of the cast is increased because of the capacity of acrylic resin to produce distortion [13].

It is anticipated that the results of this study will enhance our understanding of the open tray impression technique and aid in optimizing implant restoration procedures, ultimately benefiting patients and ensuring long-term success in implant dentistry. The aim of the present study is to compare the accuracy of four different splinting open-tray impression techniques on multiple implants.

## Methods

The present study was conducted in the Department of Prosthodontics. This in-vitro study aimed to compare the precision of four splinting materials in an open tray impression technique in multiple implant situations. Based on the material used for splinting, four groups were made (*n* = 40)- Group A: Conventional Method, Group B: Prefabricated Pattern Resin Framework, Group C: Prefabricated Metal Framework, Group D: Light Cured Pattern Resin. Initially, a rubber mold was constructed by making the index of the edentulous cast. Subsequently, melted wax was poured into the rubber mold, and the processing was performed using a heat-cured clear acrylic resin, and a master model was constructed. A pilot milling machine drill was used to drill four parallel holes in the anterior and premolar regions, which were later labeled as A, B, C, and D positions from right to left. The drilling positions were initially planned to provide equal distances between the implant analogs. Then, sequential drilling was carried out, and four 3.75‑mm diameter and 13-mm long ADIN (Adin Dental Implant Systems, Israel) implant analogs with internal hex were placed in the acrylic model using a surveyor for proper orientation. Absolute parallelism of the implant analogs in the cast was vital as it establishes a reliable baseline for measuring the deviations and assessing the precision of the different splinting materials. It also ensured that any deviations observed between the impressions and the reference model were primarily due to the splinting materials and not caused by inconsistencies in the implant analog alignment. This provided a standardized and consistent reference for evaluating the splinting materials’ precision and ensuring the findings’ clinical relevance. The impression posts were then manually screwed to the implant analogs using an open tray, and they were secured to the implants using 10 mm flat head guide pins with a 15 N.cm torque. The copings were bonded using a polyether adhesive (Polyether Adhesive, 3 M ESPE, Saint Paul, MN, USA) to reduce the impression’s movement.

**Fig. 1:**
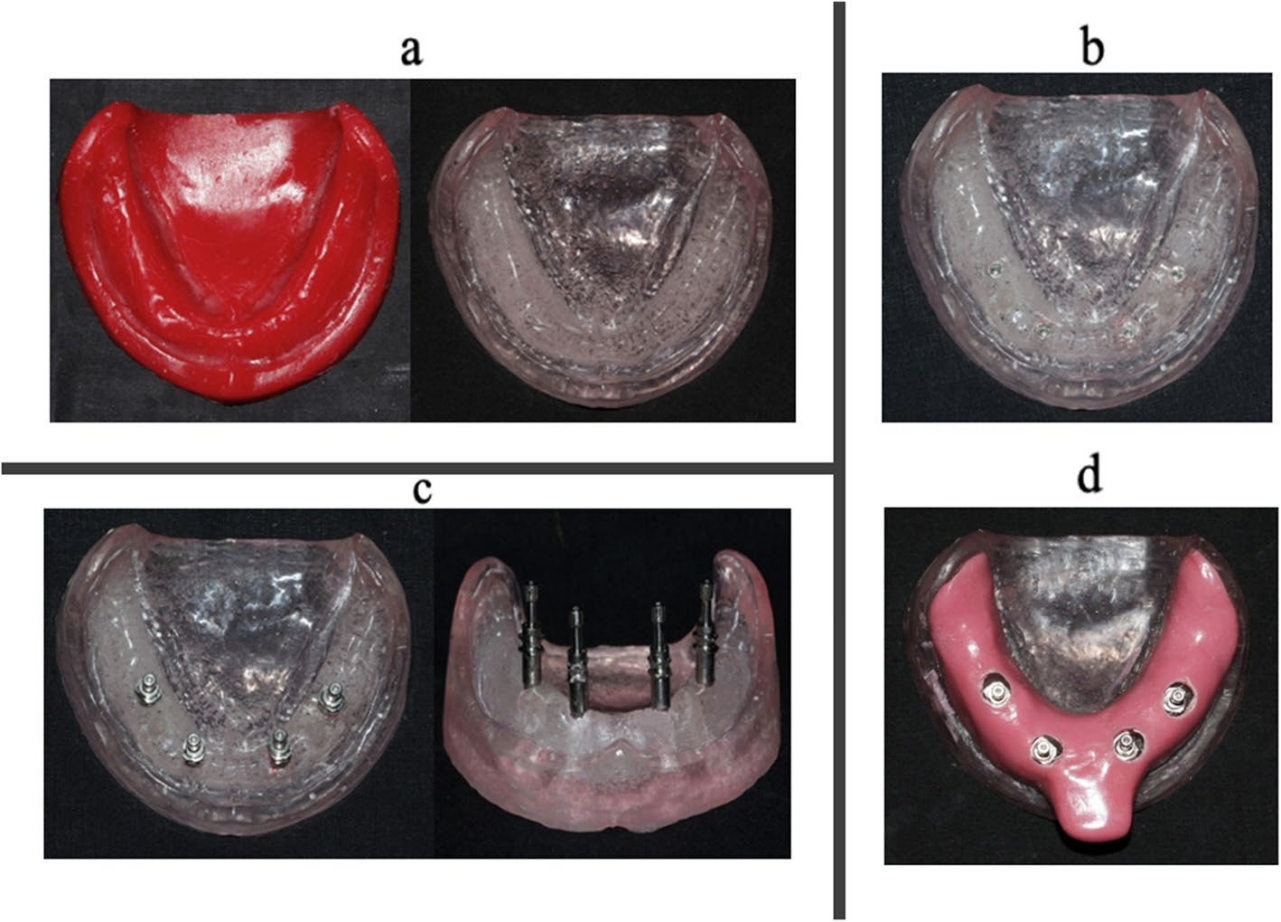
**a** The wax pattern of the master cast converted into a heat-cured clear acrylic cast; **b**- Heat-cured transparent acrylic resin master model with implant analogues numbered A to D; **c**- Impression copings attached to analogues; **d**- Custom tray fabrication with holes in **A**, **B**, **C**, **D** region

Three stops were made in the mandibular master model: one in the front and two in the back. This guaranteed that the impression trays were oriented correctly. In the master model, a two-layer wax spacer was used to provide space for the impression material, and a custom tray was made using self-curing acrylic resin. This customized tray fitted the master model and created windows for implant analogs (Fig. 1).

Forty custom trays, 10 per group, with windows in the corresponding region, were fabricated using autopolymerizing acrylic resin. For stability purposes, trays were kept undisturbed for 24 h before making an impression. Four different splinting materials, which were divided into four groups, were used to create impressions:

### Group A: conventional method

**Fig. 2:**
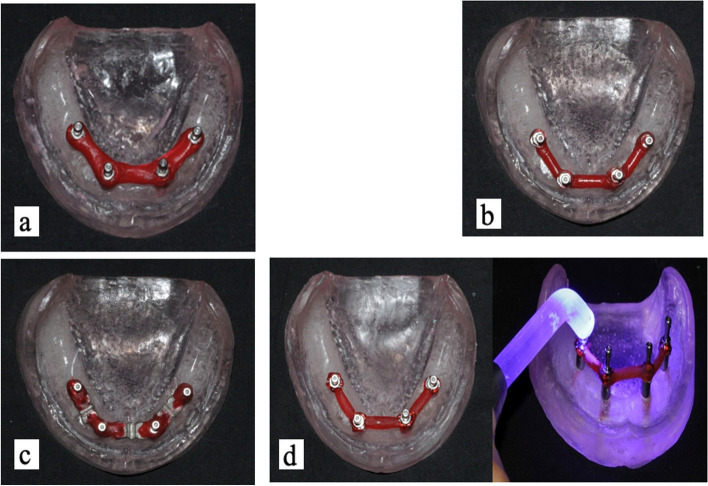
**a** The open tray impression copings were split with GC pattern resin and sectioned to account for the shrinking caused by polymerization; **b**- Open tray impression copings splinting with prefabricated pattern resin bar; **c**- Prefabricated metal framework for the splinting of the open tray impression copings; **d**- Open tray impression coping splinting with light cure pattern resin

On each open-tray impression coping, the dental floss was wrapped tightly and held in place securely. The resin (GC pattern resin; GC Corp, Tokyo, Japan) was mixed in a ceramic jar at a ratio of 2 g to 1 ml. It was subsequently encircled around the dental floss and the open tray impression posts and was left to set for 4 min. Then, these splints were cut into smaller pieces using a diamond disk in the middle of each piece to leave a standard gap of 0.2 mm between each piece. The pieces were then put back together just before the impression process with a small amount of pattern resin, attached to the splints, and left for four more minutes to harden. (Fig. 2a). This was done to minimize the resin shrinkage during polymerization.

### Group B: prefabricated pattern resin framework

I. A prefabricated pattern-resin framework (GC pattern resin) was fabricated using a silicone index made from a wire with a diameter of 3 mm. The prepared index was then poured with pattern resin. For using this framework, approximately 4 min were required after mixing. The framework was then removed from the silicon index, and the framework was ready for splinting.
II. The ends of the framework were attached to the posts using a small amount of a new pattern resin. After waiting 4 min for the fresh resin to solidify, the bar was attached to the impression posts using the Bead Brush method. (Fig. 2b).

The “Bead Brush method” is a technique used for creating resin patterns for various dental restorations, such as crowns, bridges, or dentures. The resin material is applied as small beads onto a prepared surface and then shaped and contoured using a brush. Mixing the Material: The resin material was mixed according to the manufacturer’s instructions. It involved combining a resin base and a catalyst to initiate the hardening process. Applying the resin beads: Small beads of the mixed resin material are strategically placed onto the prepared surface. Shaping and contouring: Using a brush (commonly called a bead brush or a brush with bristles that mimic resin beads), the resin material is contoured and shaped. The brush helps distribute and blend the resin beads to create the desired form and anatomy of the restoration. Curing the resin: Once the shaping and contouring is complete, the resin material is cured using a curing light or similar curing method recommended by the resin manufacturer. This hardens the resin and ensures a stable and durable resin pattern. Final adjustments and finishing: After the resin has fully cured, any excess material was trimmed and polished to achieve a smooth and natural-looking surface. The resin pattern is further refined to ensure proper fit, function, and aesthetics [14, 15].

### Group C: prefabricated metal framework

Impression posts were splinted using a prefabricated metal framework. First, the framework’s design was made in pattern resin, which was approximately 8 × 5 × 6 mm, and subsequently cast into a cobalt-chromium alloy and sandblasted. Holes were made 1 mm from the end of the framework so that dental floss was looped around the framework and the impression post. Finally, the frameworks and posts were secured with a small amount of additional resin, which was allowed to polymerize for four min before making the impression (Fig. 2c). Adding grooves to the design helped to bind the impression material rigidly to the metal framework.

### Group D: light-cured pattern resin

The attached impression posts were splinted with a light-cured pattern resin. Primopattern LC Gel and paste (Primotec, USA) were used as splinting materials. The first primopattern LC Gel was used to build up around each post and light cured. Later, the primopattern LC paste was pre-shaped into a bar form with the fingers, placed between the copings, and attached to the adjacent impression post. Light curing was performed using ultraviolet (UV light) for 3–5 min (Fig. 2d).

### Impressions-making procedure

**Fig. 3:**
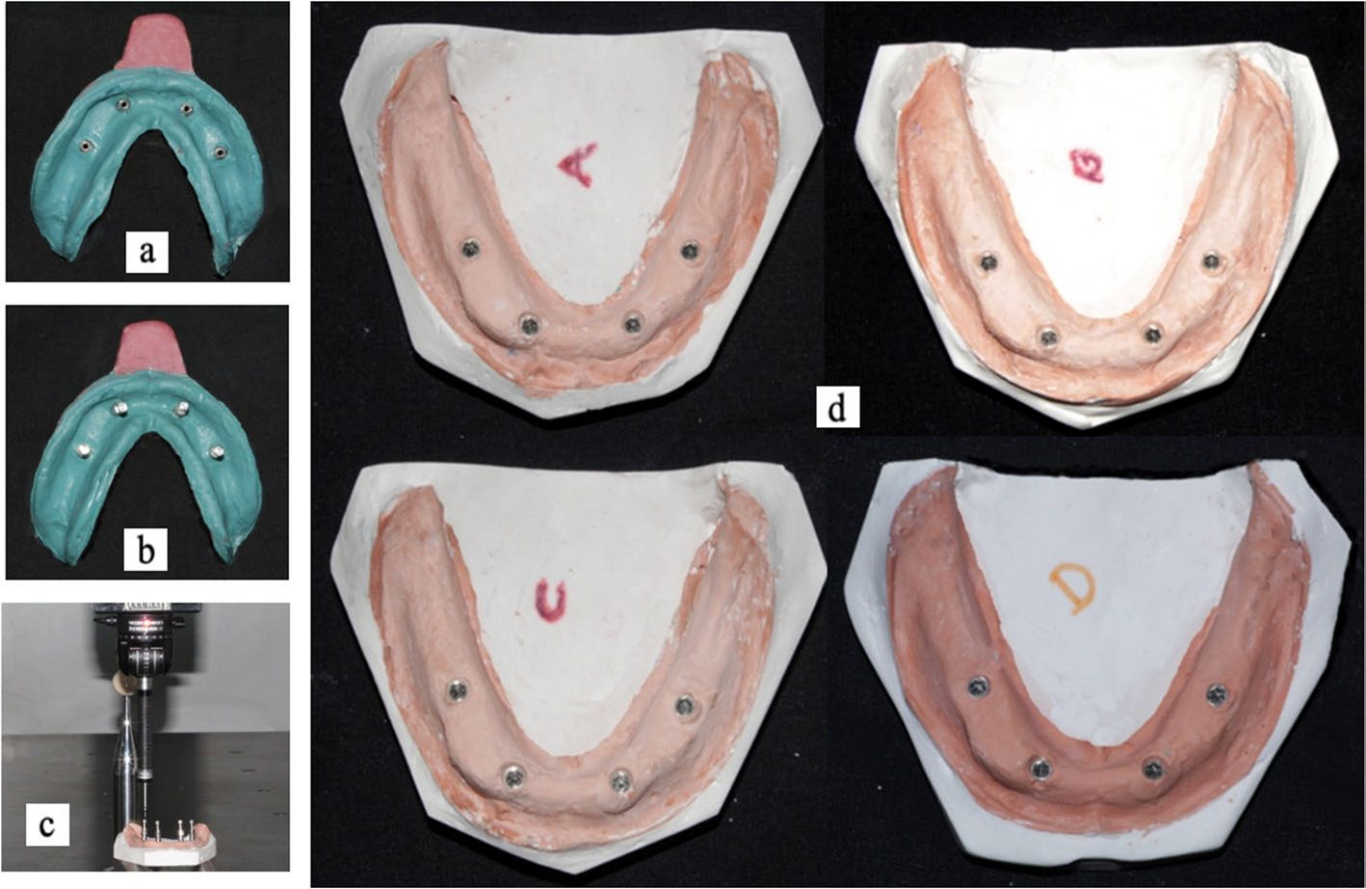
**a** The impression was made with medium body polyether; **b**- Impression coping with attached implant analogs; **c**- Measurement of distances on the control models and master casts using a coordinated measuring machine; **d**- Master casts obtained by splinting the open tray impression copings with four splinting groups. (Group A, Group B, Group C, Group D)

Custom trays are checked to fit the master model. Windows were created for the implant analogs, and a tray adhesive was applied to the custom tray. The medium body polyether was subsequently mixed in the Pentamix™ Automatic Mixing Unit (3 M ESPE, Saint Paul, MN, USA) before being loaded into the custom tray. To avoid impression errors around the impression posts, a small quantity of material was syringed around the posts. The tray was then quickly placed on the model to obtain an impression. Extra material was removed from the open-tray windows to show the guide pins. The impression was left for 6 min, as recommended by the manufacturer. After the polymerization process was completed, the guide pins of the impression posts were unfastened with a hex driver, and the trays were removed from the model. The ensuing impression, which included the impression post and guide pins, was subsequently produced. (Fig. 3a). The guiding pins were then tightened with the hex driver after the implant analog was secured to the impression post (Fig. 3b), and then the impression was poured with a type IV dental stone. Before taking measurements, all casts were removed from the impressions and kept at room temperature for at least 24 h. For each group, a total of 10 impressions were obtained. (Figure 3c and d).

**Fig. 4:**
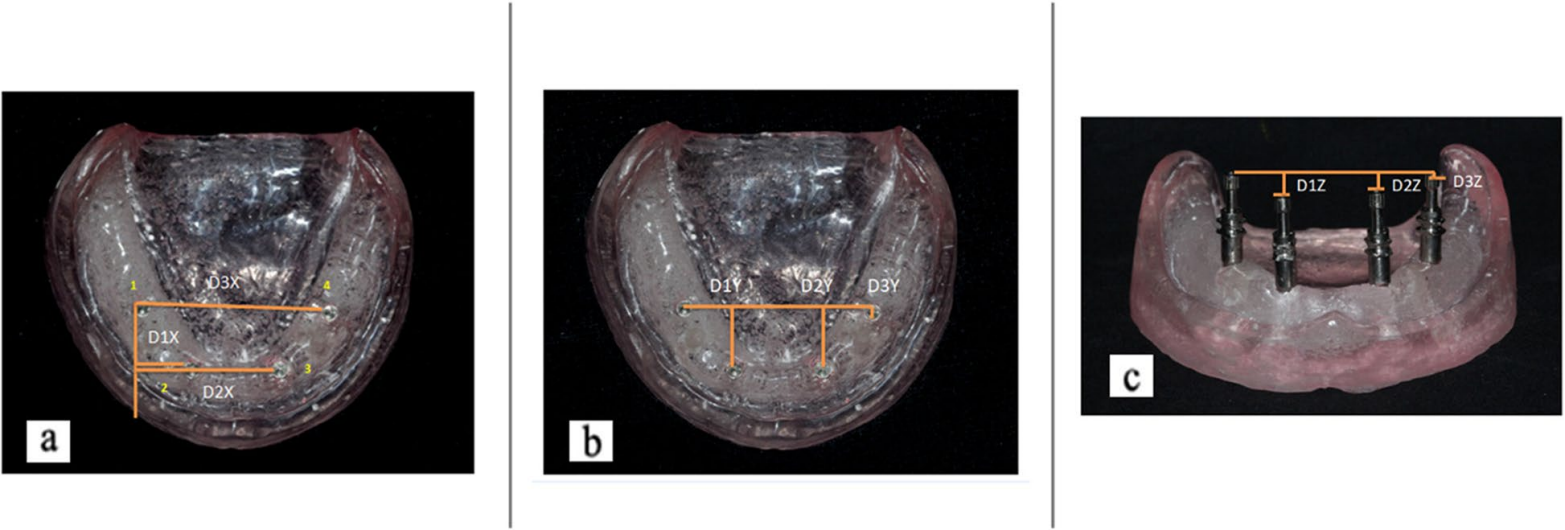
**a **Schematic representation of inter-implant distances in the X-axis; **b**- Schematic representation of inter-implant distances in the Y-axis; **c**- Schematic representation of inter-implant distances in the Z-axis

Casts were taken from all the groups. Casts were examined to determine the positional accuracy of the implant analog, and data were collected from the Coordinated Measuring Machine on the X-, Y-, and Z-axes (Fig. 4a-c).

The coordinate measurement machine (CMM) is a device commonly used in dentistry to test the placement of implants with high precision [16]. Working steps for CMM for testing the placement of implants:

1. Calibration: Before the measurements, CMM was calibrated. This was done to ensure the machine accurately interprets and records measurements.
2. Scanning the implant position: The CMM uses a probe scanner to scan the dental implant and surrounding area. The probe captured the three-dimensional coordinates of specific points around the implant.
3. Data collection: As the probe or scanner moves over the implant, it collects a series of measurements. These measurements include the X, Y, and Z coordinates of various points on the implant surface.
4. Data analysis: The CMM software processed the collected measurements once the data collection was complete. It analyzed the coordinates and calculated the exact position and orientation of the implant in relation to a reference point or coordinate system.
5. Comparison to planned placement: The CMM software compares the measured implant position to the planned or desired position, typically based on the surgical guide. It calculates any deviations or discrepancies between the actual and planned implant positions.
6. Evaluation and adjustment: The implant placement was evaluated based on the comparison results.

The CMM provides highly accurate and detailed information about implant placement, ensuring precise positioning.

Statistical analyses were performed using the Kruskal-Wallis test and the posthoc test. Inferences were drawn from the obtained data and are discussed below. A Microsoft Office Excel document (version 2019, Microsoft Redmond Campus, Redmond, Washington, United States) was used to compile the data. IBM’s SPSS version 26.0 was used to analyze the collected data statistically.

## Results

**Fig. 5:**
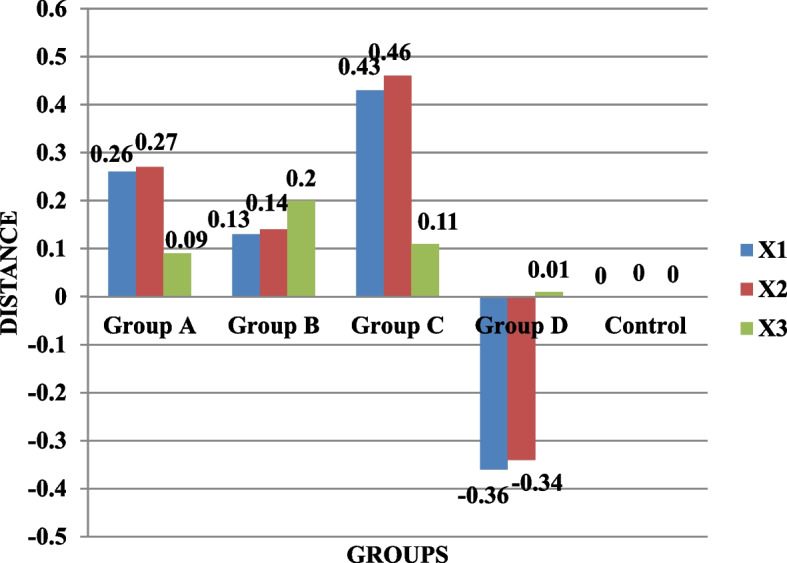
Comparison of the four splinting materials’ mean deviation distances (µm) for X1, X2, and X3

**Fig. 6:**
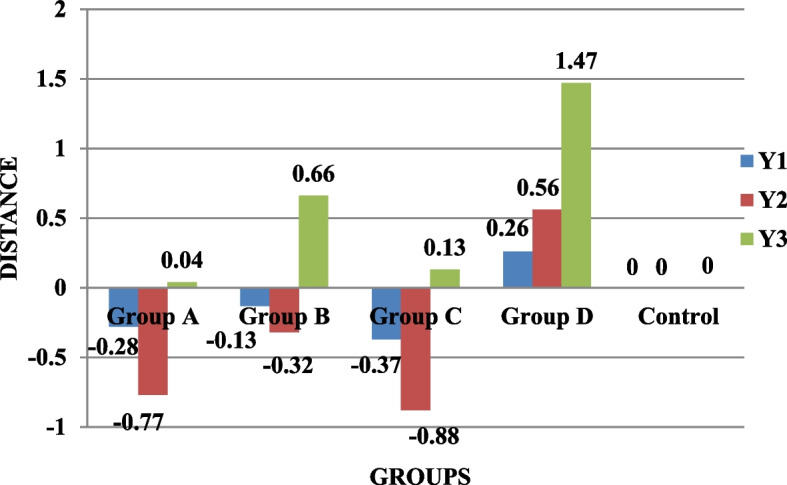
Comparison of X1, X2, and X3 mean deviation distances (µm) for the four splinting materials

**Fig. 7:**
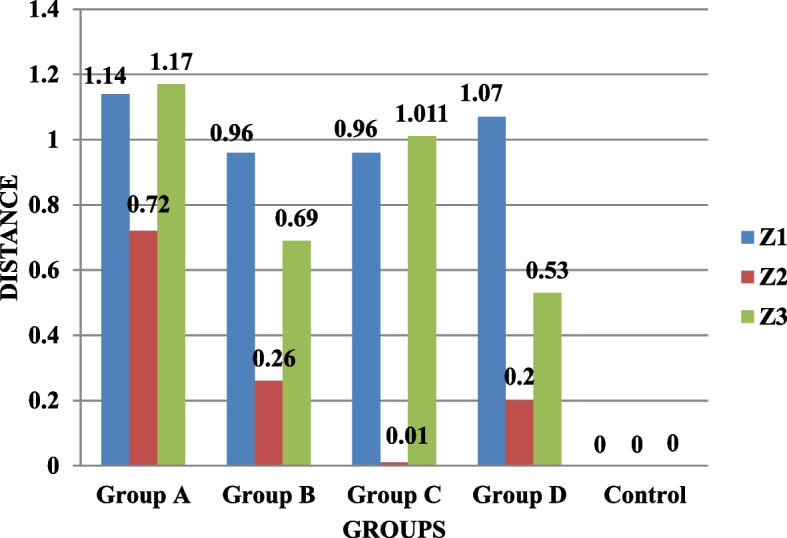
Comparison of the mean deviation distances (µm) of the four splinting materials for Z1, Z2, and Z3

The results showed that in the distances X1, X2, and X3, there was a statistically significant difference (*p* < 0.05) between the groups. For the deviation distance X3, there was a statistically significant difference (*p* < 0.05) across groups; however, it was non-significant for the deviation distances X1 and X2. The comparison between groups revealed a statistically significant difference (*p* < 0.05) for distance Y1 but not for distances Y2 and Y3. For the deviation distances Y1, Y2, and Y3, there was a statistically significant difference (*p* < 0.05) between the groups. For distances Z2 and Z3, there was a statistically significant difference (*p* < 0.05) between the groups, whereas the results for distance Z1 were non-significant. The deviation distance Z1 had a high statistically significant difference between the groups (*p* < 0.05); however, the findings for distances Z2 and Z3 were non-significant. All groups were compared using the Kruskal-Wallis test. For distances X1 (0.025), X2 (0.028), X3 (0.019), Y2 (0.30), Y3 (0.037), and Z1 (0.001), the results were statistically significant, whereas for distances Y1 (0.051), Z2 (0.197), and Z3 (0.065), the results were insignificant (Table 1; Figs. 5, 6 and 7).

**Table 1:** Comparison of mean distances (µm) for X-, Y-, and Z-axis using the Kruskal-Wallis test

Groups	X1		Y1		Z1		X2		Y2		Z2		X3		Y3		Z3	
	Mean	S.D.	Mean	S.D.	Mean	S.D.	Mean	S.D.	Mean	S.D.	Mean	S.D.	Mean	S.D.	Mean	S.D.	Mean	S.D.
**A**	10.9	0.6	13.25	0.5	1.27	0.9	29.9	0.6	13.14	1.4	1.55	0.9	40.8	0.14	1.38	1.3	1.65	1.1
**B**	10.7	0.8	13.4	0.7	1.08	1	29.7	0.9	13.58	2	1.09	0.6	41	0.22	2	1.8	1.17	0.8
**C**	11	0.6	13.16	0.5	1.08	0.8	30.1	0.6	13.03	1.4	0.84	0.8	40.9	0.08	1.47	1.2	1.49	1.2
**D**	10.2	0.8	13.79	0.7	1.19	0.7	29.2	0.8	14.48	1.6	1.03	0.4	40.8	0.13	2.81	0.7	1.01	0.7
**Control**	10.6	0	13.53	0	0.12	0	29.6	0	13.91	0	0.83	0	40.7	0	1.34	0	0.48	0
**F**	11.146		18.359		9.443		10.874		6.03		10.68		11.9		8.839		10.209	
***P*** **value **	0.025*		0.001**		0.051#		0.028*		0.197#		0.030*		0.019*		0.065#		0.037*	

*statistically significant difference (*p* < 0.05), **statistically highly significant difference (*p* < 0.01), #non-significant difference (*p* > 0.05)

Furthermore, for pairwise comparisons, Post Hoc Tukey’s test was used. The results were statistically significant only for the distance X3 when a comparison was performed for Group B vs. D (*p* = 0.27) and Group B vs. control (*p* = 0.014). In contrast, the results were non-significant for the rest of the comparisons (*p* > 0.05). The results were statistically insignificant for the pairwise comparisons for the distances Y1, Y2, and Y3 (*p* > 0.05). The results were statistically significant for the distance Z1 comparisons, namely, control vs. Group A (*p* = 0.012), control vs. Group B (*p* = 0.049), control vs. Group C (*p* = 0.048) and control vs. Group D (*p* = 0.021), and for the distance Z3 comparison for control vs. Group A (*p* = 0.033). The results were statistically insignificant for the distance Z2 comparisons (*p* > 0.05) (Table 2).

**Table 2:** Pairwise comparison between Post Hoc Tukey’s Test for distances on X-, Y-, and Z-axis

Dependent Variable	(I) GROUPS	(J) GROUPS	Mean Difference (I-J)	*P* value	Mean Difference (I-J)	*P* value	Mean Difference (I-J)	*P* value
		X			Y		Z	
**1**	**A**	B	0.13	0.990#	-0.14	0.977#	0.18	0.980#
		C	-0.16	0.975#	0.09	0.996#	0.18	0.981#
		D	0.63	0.188#	-0.54	0.218#	0.07	0.999#
		Control	0.26	0.879#	-0.28	0.799#	1.14	0.012*
	**B**	A	-0.13	0.990#	0.14	0.977#	-0.18	0.980#
		C	-0.3	0.824#	0.23	0.880#	0	1.000#
		D	0.5	0.410#	-0.39	0.524#	-0.11	0.997#
		Control	0.13	0.989#	-0.13	0.983#	0.96	0.049*
	**C**	A	0.16	0.975#	-0.09	0.996#	-0.18	0.981#
		B	0.3	0.82#	-0.23	0.880#	0.01	1.000#
		D	0.8	0.052#	-0.63	0.107#	-0.1	0.998#
		Control	0.43	0.545#	-0.37	0.586#	0.96	0.048*
	**D**	A	-0.63	0.188#	0.54	0.218#	-0.07	0.999#
		B	-0.5	0.410#	0.39	0.524#	0.11	0.997#
		C	-0.8	0.052#	0.63	0.107#	0.11	0.998#
		Control	-0.36	0.701#	0.26	0.837#	1.07	0.021*
	**Control**	A	-0.26	0.879#	0.28	0.799#	-1.14	0.012*
		B	-0.13	0.989#	0.13	0.983#	-0.96	0.049*
		C	-0.43	0.545#	0.37	0.586#	-0.96	0.048*
		D	0.36	0.701#	-0.26	0.837#	-1.07	0.021*
**2**	**A**	B	0.13	0.991#	-0.44	0.956#	0.45	0.527#
		C	-0.18	0.965#	0.11	1.000#	0.7	0.134#
		D	0.62	0.209#	-1.34	0.235#	0.51	0.405#
		Control	0.27	0.866#	-0.77	0.742#	0.72	0.120#
	**B**	A	-0.13	0.991#	0.44	0.956#	-0.45	0.527#
		C	-0.31	0.801#	0.55	0.904#	0.24	0.918#
		D	0.49	0.434#	-0.89	0.626#	0.05	1.000#
		Control	0.14	0.985#	-0.32	0.985#	0.26	0.899#
	**C**	A	0.18	0.965#	-0.11	1.000#	-0.7	0.134#
		B	0.31	0.801#	-0.55	0.904#	-0.24	0.918#
		D	0.81	0.052#	-1.45	0.169#	-0.18	0.969#
		Control	0.46	0.489#	-0.88	0.634#	0.015	1.000#
	**D**	A	-0.62	0.209#	1.34	0.235#	-0.51	0.405#
		B	-0.49	0.434#	0.89	0.626#	-0.05	1.000#
		C	-0.81	0.052#	1.45	0.169#	0.18	0.969#
		Control	-0.34	0.752#	0.56	0.899#	0.2	0.958#
	**Control**	A	-0.27	0.866#	0.77	0.742#	-0.72	0.120#
		B	-0.14	0.985#	0.32	0.985#	-0.26	0.899#
		C	-0.46	0.489#	0.88	0.634#	-0.01	1.000#
		D	0.34	0.752#	-0.56	0.899#	-0.2	0.958#
**3**	**A**	B	-0.1	0.413#	-0.61	0.772#	0.47	0.737#
		C	-0.02	0.997#	-0.09	1.000#	0.15	0.994#
		D	0.08	0.673#	-1.42	0.074#	0.63	0.482#
		Control	0.09	0.517#	0.04	1.000#	1.17	0.033*
	**B**	A	0.1	0.413#	0.61	0.772#	-0.47	0.737#
		C	0.08	0.616#	0.52	0.857#	-0.31	0.925#
		D	0.19	0.027*	-0.8	0.560#	0.16	0.994
		Control	0.2	0.014*	0.66	0.722#	0.69	0.392
	**C**	A	0.02	0.997#	0.09	1.000#	-0.15	0.994
		B	-0.08	0.616#	-0.52	0.857#	0.31	0.925
		D	0.1	0.468#	-1.33	0.107#	0.47	0.736
		Control	0.11	0.327#	0.136	0.999#	1.02	0.087
	**D**	A	-0.083	0.673#	1.42	0.074	-0.63	0.482
		B	-0.19	0.027*	0.8	0.56	-0.16	0.994
		C	-0.1	0.468#	1.33	0.107	-0.47	0.736
		Control	0.01	0.999#	1.47	0.06	0.53	0.646
	**Control**	A	-0.09	0.517#	-0.04	1	-1.17	0.033
		B	-0.2	0.014*	-0.66	0.722	-0.69	0.392
		C	-0.11	0.327#	-0.13	0.999	-1.01	0.087
		D	-0.01	0.999#	-1.47	0.06	-0.53	0.646

*statistically significant difference (*p* < 0.05), **statistically highly significant difference (*p* < 0.01), #non-significant difference (*p* > 0.05)

## Discussion

A favorable outcome in implant prosthodontics can only be attained appropriately when passively fitting prostheses are created [17]. The master cast from a reproducible impression must match the clinical condition. Direct techniques have been shown to be more accurate than indirect techniques in most scientific studies [17]. Splinting may stabilize impression copings under stress for analog tightening and limit the rotational mobility in resilient impression materials. Splinting plays a crucial role in casting accuracy regardless of the impression material [18].

This study evaluated and compared the effectiveness of autopolymerizing pattern resins, prefabricated pattern resin bars, prefabricated metal frameworks, and light-cure pattern resins as splinting materials. Polyether is viable for edentulous patients with several implants supporting a prosthesis. Polyether (Monophase) was utilized as the impression material because it is done in a one-step technique with materials of medium viscosity to allow the material itself to record finer details while preventing the material in the tray from sagging; it takes less time, and it is comparatively simple to perform [18].

The impression copings were secured to the implants at a 15 N.cm torque before obtaining impressions. To decrease the movement of the impression, the copings were bonded with a polyether adhesive. The torque wrench was not used to tighten the implant replicas to the impression copings to avoid rotation [19]. Type IV dental stone was used to pour the casts following the manufacturer’s guidelines.

The rotation of impression copings in the set material during implant analog fastening is a disadvantage of the direct impression technique. Therefore, changes in dimensions are possible in any given direction. The inter-implant distances were measured along the x-, y-, and z-axes so that the size of the inaccuracy that can occur in three dimensions can be studied. All casts were measured using a coordinate measuring machine [20] that was accurate within 5 μm. In absolute distortion analysis, an external reference point is utilized as a reference to compute distortion, but in relative distortion analysis, one implant or replica is used as a reference to calculate distortion. As the prosthesis connects each implant to the others, the stress exerted on the implants depends on their locations with respect to one another. This study measured the distances between implants to conduct a relative distortion analysis.

Many other factors affect the behavior of transfer splinting during implant impression. One of the main factors is the polymerization shrinkage. As in group A, a large volume of resin was used to create the splint, which could be associated with the significant polymerization shrinkage reported in the literature, which ranges from 6.5 to 7.9% for acrylic resin [20]. This technique produced more strain on the acrylic resin and impression post interface, subsequently affecting the transfer accuracy. Previous studies have established the efficacy of sectioning and re-joining resins to compensate for this strain [21]. Cabral and Guedes [22] proposed a method of compensation that entails cutting the splint in half 17 min after it was set and joining it back with the same resin before taking the impression.

The results of the present study showed that the light-cure pattern resin and metal framework can be used as an alternative splinting material for the accurate reproduction of spatial relationships. The prefabricated pattern resin bar produced the most precise results because of the low shrinkage of the pattern resin and splinting method. Splints were fabricated using a technique described in the literature [23], with slight modifications, in which bars of acrylic resin were made with the aid of the putty index of metal wire instead of plastic straws with a cross-section of approximately 3 mm. It has been established that all acrylic resins exhibit polymerization shrinkage. Moon et al. [13] found that 80% of polymethyl methacrylate (PMMA) polymerization shrinkage occurs within 17 min at room temperature, and no substantial shrinking occurs beyond 24 h. Therefore, the effect of polymerization shrinkage of the pattern resin was minimized by using a pattern resin bar that was fabricated 24 h before the splinting procedure [11]. Therefore, the only shrinkage that would affect the accuracy was the shrinkage of the added resin used to connect the prefabricated bar to the impression post. Only a small amount of additional resin was used to join the bars and impression posts. In this study, the transfer of resin bars with the addition of acrylic resin provided cast models with high precision. This finding is consistent with previous studies [23–28].

There is a paucity of information on the efficacy of light-cure pattern resins and metal framework bars as splinting materials because they have not been extensively investigated. The outcomes of the materials used in this study were compared to those of the control group. As a result, the range of changes in the metal framework splinted group, i.e., group C in the X axis with mean values were D1x-11.04 μm, D2x-30.05 μm, and D3x-40.86 μm while in the group D, (D1x-10.23 μm, D2x-29.24 μm, D3x-40.76 μm) was nearly in the same range as the mean values of the control group (D1x- 10.60 μm, D2x -29.59 μm, D3x -40.74 μm). Additionally, for group C (light cure pattern resin), the values were (D1y-13.16 μm, D2y-13.03 μm, and D3y-1.47 μm), and for group D (light-cured pattern resin), the values were (D1y-13.79 μm, D2y-14.48 μm, D3y-2.81 μm). These values were close to the control group (D1y-13.53 μm, D2y-13.91 μm, D3y-1.34 μm). Mean values in the Z axis for Group C were (D1z-1.08 μm, D2z-0.84 μm, D3z- 1.49 μm) and for Group D were (D1z-1.19 μm, D2z-1.03 μm, D3z- 1.01 μm) while the mean values for the control group were (D1z-0.12 μm, D2z-0.83 μm, D3z- 0.48 μm). The differences might be ascribed to the hardness of the splinting materials employed to limit the vertical movement of copings during implant replica attachment to the impression coping [29].

In this study, deviation in Groups A, B, C, and D was not statistically significant in the D1x and D2x axes. (D1x, *p* = 0.8, *p* = 0.98, *p* = 0.54, *p* = 0.70, and D2x, *p* = 0.98, *p* = 0.86, *p* = 0.48, *p* = 0.75) because the obtained values showed minimum deviation from the control group. In the D3x axis, less deviation was observed in group D (*p* = 0.99), whereas group B showed the greatest deviation (*p* = 0.14). When assessed against the other groups, group B had the lowest deviation on the D1y axis (*p* = 0.98), whereas group C had the most (*p* = 0.58). Similarly, in the D2y axis, the minimum deviation was observed in group B (*p* = 0.98), and group C showed more deviation from the control group (*p* = 0.63). Group A showed less deviation (*p* = 1.00), whereas Group D showed the greatest deviation (*p* = 0.06) on the D3y axis. However, a more statistically significant deviation was observed on the D1z axis. Group A showed the greatest deviation (*p* = 0.12), while Group B showed less (*p* = 0.49). On the D2z axis, group C showed the least deviation (*p* = 1.00), whereas group A showed the greatest deviation (*p* = 0.12). On the D3z axis, group D showed the least deviation (*p* = 0.64), whereas group A showed the greatest deviation (*p* = 0.03). The obtained values were used to gauge the precision of the splinting materials. Based on the data, the interpretation of the research is affected using polyether adhesive, polyether impression material, the rigidity of the splinting materials, tolerance between implant components, and torque used to secure the implant replica that could determine the magnitude of distortion [30].

Lee et al. [31] and Papaspyridakos et al. [32] found that using a splint technique resulted in more accurate implant impressions than a non-splint technique. The outcomes of this study, which employed the splint technique, are related to one another.

In a study performed by Lee et al. [29] on the reliability of implant impression procedures and the effect of splinting materials and methods, it became apparent that splinting the impression copings with autopolymerizing resin after compensating for polymerization shrinkage could enhance master cast accuracy and can be used as an efficient splinting material for implant impression protocols.

The light-cure pattern resin is urethane-methacrylate resin (UDMA), which has less polymerization shrinkage than PMMA acrylic resin and does not contain methyl methacrylate monomer or peroxide. This new material is ready to use, has one component unit, and is thixotropic in nature. It comes in gel and paste forms, which are useful for precise application, stay in place, do not run, and are very economical. This new material is dimensionally stable and has high surface hardness and strength. Curing can be performed using a conventional light-curing unit or gun (320–500 nm wavelength). The result obtained using light cure pattern resin as splinting material was comparable to that of Group A and Group B. The result of this study was like the study conducted by Papaspyridakos et al. [33]. Gibbs et al. [34] investigated the polymerization shrinkage of photopolymerizing and autopolymerizing pattern resin. They determined that photopolymerizing gel and autopolymerizing pattern resin had comparable shrinkage values, whereas the shrinkage of light-curing paste was substantially greater.

The strengths of the study include (1) Controlled environment: The study was conducted in an in vitro setting, which allowed for strict control over the variables and eliminated potential confounding factors that could arise in a clinical setting; (2) Multiple implant evaluation: The study assesses the relative positioning accuracy of multiple implants, providing valuable insights into the performance of the different splinting materials; (3) Clear methodology: The methodology is described in sufficient detail, including the materials used, the construction of the model, and the placement of implants. This allows for replication and a better understanding of the study. (4) Statistical analysis: The study utilizes statistical analysis to evaluate the results, providing quantitative evidence to support the findings and determine the significance of the observed differences.

On the other hand, the limitations could be mentioned as (1) Lack of clinical relevance: While the study aims to simulate the clinical environment, the findings may not directly translate to actual clinical outcomes. In vitro studies cannot fully replicate the complexities and variations present in a patient’s oral cavity. Additionally, the clinical application of using metal may not be feasible, but the idea of using metal splinting was to use a stiff material and compare it with other materials being used; (2) Limited sample size: A larger sample size would enhance the study’s reliability; (3) Limited scope of splinting materials: The study only evaluates four specific types of splinting materials. Not all materials available in clinical practice are considered, limiting the broader applicability of the findings; (4) No long-term evaluation: The study focuses on the relative positioning accuracy of the implants immediately after placement. Long-term stability, osseointegration, and clinical outcomes are not assessed.

Overall, this study provides valuable insights into the relative positioning accuracy of different splinting materials in an in vitro setting. However, the findings should be interpreted cautiously due to the abovementioned limitations. Further research, including clinical trials with larger sample sizes, must validate these findings and explore their clinical implications.

## Conclusion

All splinting materials produced master casts with measurements close to the reference model, and all had clinical limitations. The present study found that using prefabricated pattern resin bars for splinting impression copings yielded higher accuracy levels than other materials. The latest splinting strategies utilizing light-cure pattern resin and prefabricated metal framework demonstrated comparable accuracy.

## Data Availability

The authors confirm that the data supporting the findings of this study is available upon request from the corresponding authors.

## Abbreviations

PE: Polyether
CMM: Coordinate measurement machine
PMMA: Polymethyl methacrylate
UDMA: Urethane-methacrylate
